# Genome sequence of prodigiosin-producing and tetracycline-resistant *Serratia marcescens* (IFSMLMEK1), identified from a freshwater bivalve *Lamellidens marginalis*

**DOI:** 10.1128/mra.01115-24

**Published:** 2025-03-05

**Authors:** Debasmita Mohanty, Mala Kumari, Vikash Kumar, Punam Kumari, Bhaskar Behera, Basanta Kumar Das

**Affiliations:** 1 Aquatic Environmental Biotechnology Department, ICAR-Central Inland Fisheries Research Institute 235412 , Barrackpore, West Bengal, India; 2 Department of Bioscience and Biotechnology, Fakir Mohan University 209042 , Balasore, Odisha, India; Portland State University, Portland, Oregon, USA

**Keywords:** *Serratia marcescens*, *Lamellidens marginalis*, tetracycline-resistant, prodigiosin-producing

## Abstract

We report the genome sequence of a tetracycline-resistant *Serratia marcescens*, IFSMLMEK1, isolated from the freshwater bivalve *Lamellidens marginalis*. The genome (5,185,668 bp, G+C% 59.65) reveals antibiotic resistance determinants, including tetracycline efflux genes, providing valuable insights into prodigiosin production and tetracycline resistance in environmental isolates.

## ANNOUNCEMENT

Multidrug-resistant *Serratia* species are a rising issue for healthcare and aquaculture (1). A study was carried out in India, where *Serratia marcescens* has been recognized as a pathogen associated with freshwater ornamental fish. The freshwater bivalve *Lamellidens marginalis* was taken from a sewage-fed wetland (22°32′06.6″N 88°26′13.8″ E) in West Bengal in June 2024 to isolate *S. marcescens*. The antibiotic resistance patterns of these bacteria were investigated as they pose risks when bivalves are consumed raw or undercooked due to potential contamination (2, 3).

The strain IFSMLMEK1 was isolated using the aerobic plate count method. The sample used was 1 mL of homogenate prepared from the whole tissue of the bivalve, which was inoculated into nutrient broth and subsequently transferred to nutrient agar for incubation at 30°C for 24 hours (4). A colony with pure red pigmentation was picked for identification through Gram staining, biochemical tests, and 16S rRNA gene sequencing using primers 8F and 1492R (5). Molecular identification was confirmed by comparing the sequence (PP204294.1) to those in GenBank. Antimicrobial susceptibility testing of the strain IFSMLMEK1 was conducted according to CLSI guidelines (M100-S28, 2018), taking beta-lactam antibiotics and non-beta-lactam antibiotics of different classes. These included aminoglycosides, fluoroquinolones, and tetracycline and identified as this strain was resistant to the tetracycline antibiotic.

**Fig 1:**
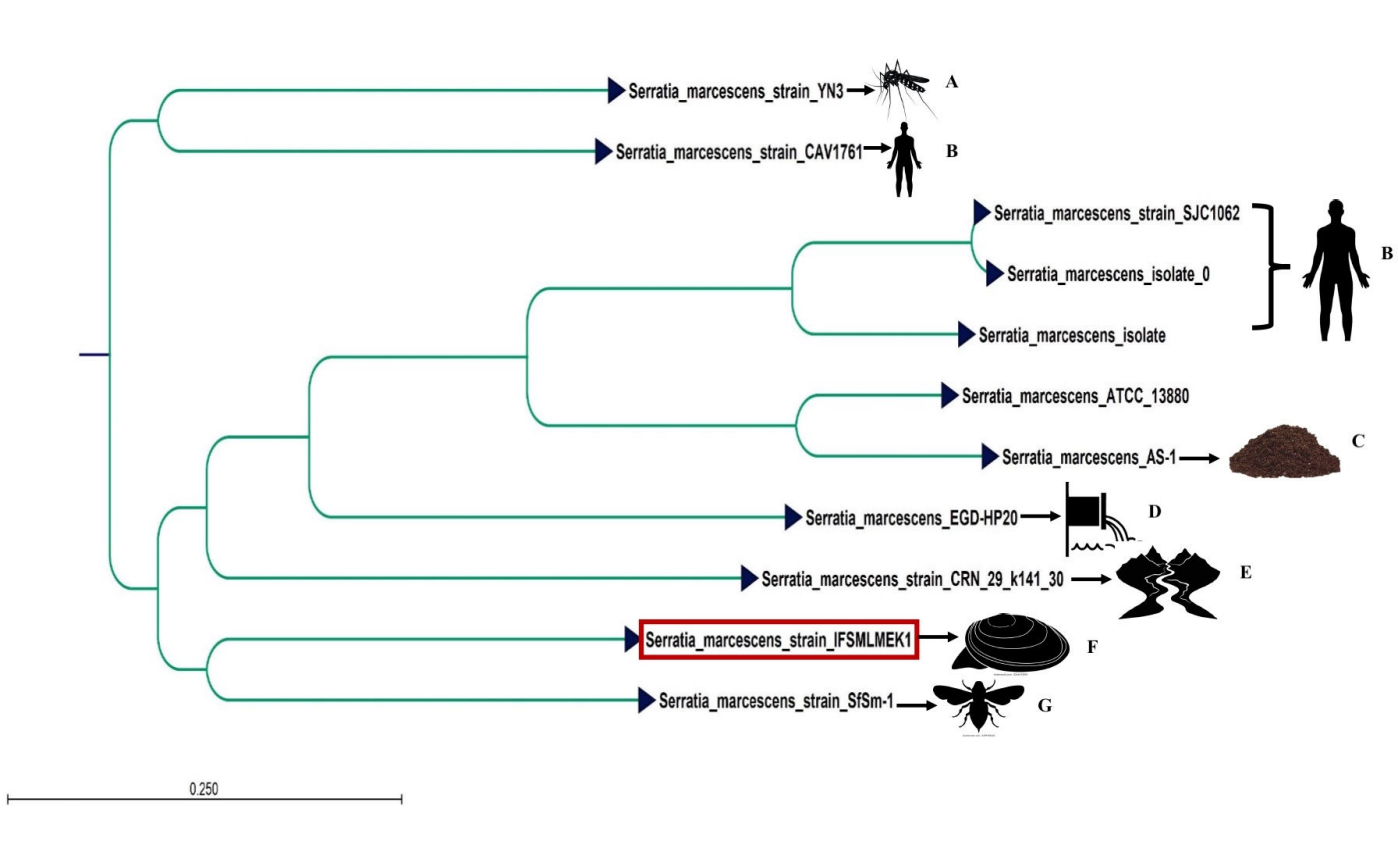
Phylogenetic analysis of IFSMLMEK1 strain (see text for methods) with other submitted whole-genome sequences of *S. marcescens* from various sources. A, *Anopheles sinensis*; B, Homo sapiens; C, sediment; D, tannery wastewater; E, water; F, *Lamellidens marginalis*; and G, *Phyllotreta striolata*.

The colonies were scraped, suspended in TE buffer, and extracted genomic DNA using the Qiagen DNeasy PowerSoil Pro Kit (47014). DNA size distribution (Agilent FEMTO pulse, Agilent), DNA quality check (NanoDrop spectrophotometer and Qubit 3.0 Fluorometer, Thermo Fisher Scientific), DNA shearing (Megaruptor 3, Belgium), sheared DNA and SMRTbell library size distribution (Agilent FEMTO pulse, Agilent), SMRTbell library preparation, SMRT library purification, library size selection to remove <5 KB fragments, and preparation of bound complex (Pacific Biosciences) were performed for sequencing. *De novo* whole-genome sequencing was performed on the PacBio Sequel II (Nucleome Informatics Pvt Ltd, Pacific Biosciences, USA) platform, generating HiFi reads using CCS (v6.2.0), which were assembled *de novo* with Flye v2.9.3 into one large contig. The genome was annotated with the Prokaryotic Genome Annotation Process (PGAP) (6). Average nucleotide identity analysis by PGAP (7) with the reference genome *Serratia marcescens* subsp. *marcescens* ATCC 13880 revealed a genomic similarity of 96.98%. GeneMarkS-2+ then makes ab initio coding region predictions for genomic regions that lack HMM or protein evidence and selects start sites for ORFs whose evidence comes from HMMs. Gene identification and annotation were done with Prokka. The completeness and contamination percentages checked by quality analysis of NCBI CheckM analysis (v1.2.3) were 97.67 and 5.06. Antimicrobial resistance genes were identified using the Resistance Gene Identifier tool in the Comprehensive Antibiotic Resistance Database (7). Additionally, IFSMLMEK1 harbors chimeric tetracycline resistance genes, including tet(O/32/O) and tet(W/N/W), contributing to a broader resistance profile. The details of the sequence data are represented in Table 1. A whole-genome phylogeny was constructed using CLC Genomics Workbench 12. The k-mer-based tree was built using a neighbor-joining approach, and Mahalanobis’ methods measured the evolutionary distance (Fig. 1).

**TABLE 1:** Detailed information of the *S. marcescens* (IFSMLMEK1) complete genome sequence

Bacterial isolate	*Serratia marcescens* IFSMLMEK1
Sequencing method	PacBio Sequel
Assembly method	Flye v2.9.3
Bases sequences (HiFi reads)	36960
Total Mb of assembled genome	5.19 Mb
No. of contig	1
Length of contig, total length (bp), and N50	5185668
rRNA operons	22
tRNA genes	91
tmRNA	1
Genome coverage	59.65×
Protein-coding	4770
Sequencing coverage	60.49
Antimicrobial resistance genes (total)	15 strict
Average nucleotide identity (ANI)	96.98%
Assembly coverage	90.74%
Type assembly coverage	91.21%
Biosample accession no.	SAMN43374428
Bioproject no.	PRJNA1152721
Accession number	CP169392.1

## Data Availability

The Whole Genome Shotgun project for *Serratia marcescens* has been deposited in GenBank under the accession number CP169392.1. The associated Sequence Read Archive (SRA) is available under SRA accession SRS22838919. This work is part of BioProject PRJNA1152721 and corresponds to BioSample SAMN43374428.
